# Anesthesia and Analgesia in the Patient with an Unstable Shoulder

**DOI:** 10.2174/1874325001711010848

**Published:** 2017-08-31

**Authors:** Ismael Acevedo Bambaren, Fernando Dominguez, Maria Elena Elias Martin, Silvia Domínguez

**Affiliations:** Ramón y Cajal Hospital. Anesthesia and Intensive Care Department. Madrid. Spain

**Keywords:** Anesthesia management, Regional anesthesia, Shoulder instability anesthesia, Single nerve blockade, Continuous nerve blockade, Intraoperative positions during shoulder arthroscopic

## Abstract

**Introduction:**

The patient with an unstable shoulder represents a challenge for the anesthesiologist. Most patients will be young individuals in good health but both shoulder dislocation reduction, a procedure that is usually performed under specific analgesia in an urgent setting, and instability surgery anesthesia and postoperative management present certain peculiarities.

**Material and Methods:**

For the purpose of the article, 78 references including clinical trials and reviews were included. The review was organized considering the patient that presents an acute shoulder dislocation and the patient with chronic shoulder instability that requires surgery. In both cases the aspects like general or regional anesthesia, surgical positions and postoperative pain management were analyzed.

**Conclusion:**

The patient with an acutely dislocated shoulder is usually managed in the emergency room. Although reduction without analgesia is often performed in non-medical settings, an appropriate level of analgesia will ease the reduction procedure avoiding further complications. Intravenous analgesia and sedation is considered the gold standard but requires appropriate monitorization and airway control. Intraarticular local analgesic injection is considered also a safe and effective procedure. General anesthesia or nerve blocks can also be considered. The surgical management of the patient with shoulder instability requires a proper anesthetic management. This should start with an exhaustive preoperative evaluation that should be focused in identifying potential respiratory problems that might be complicated by local nerve blocks. Intraoperative management can be challenging, especially for patients operated in beach chair position, for the relationship with problems related to cerebral hypoperfusion, a situation related to hypotension events directly linked to patient positioning. Different nerve blocks will help attaining excellent analgesia both during and after the surgical procedure. An interescalene nerve block should be considered the best technique, but in certain cases, other blocks can be considered.

## 1 ANESTHESIA AND ANALGESIA FOR THE PATIENT WITH AN ACUTE SHOULDER DISLOCATION

### 1.1 Introduction

Anesthetic techniques in shoulder surgery have evolved along with surgery. The performance of increasingly complex but less invasive techniques in the unstable shoulder has required the development of anesthetic techniques that allow an adequate control pain during the intraoperative and postoperative period. There are two clinical situations in the patient with an unstable shoulder: the patient with an acutely dislocated shoulder and the patient with recurrent shoulder instability. In the first situation the priority is to reduce and stabilize the shoulder Joint and not the surgical repair, being general anesthesia (GA) or sedation the first anesthetic choice. In the patient with shoulder instability that requires surgery the priority is to provide conditions that lead a safe articular fix and regional anesthesia (RA) can be considered a complement of anesthetic management. In this respect, all nerve blockades of the brachial plexus (BP) used in the shoulder surgery, like the Interescalene nerve block (ISNB), supraclavicular nerve block (SCNB), suprascapular nerve block (SSNB), will be considered in this review.

## 2 ANESTHETIC MANAGEMENT OF THE PATIENT WITH CHRONIC SHOULDER INSTABILITY THAT REQUIRES SURGERY

The patient with a dislocated shoulder probably epitomizes the acute trauma case: a usually young patient with severe pain and disability that requires prompt management. As such, many different medical personnels are usually involved in its diagnosis and management. Closed reduction by different methods is usually the best possible option and, irrespective of the particular technique used, this is often a painful procedure. Different anesthetic options should be considered when encountered with these patients:

### 2.1 Reduction Without Analgesia

This is probably the most frequently alternative used in a non-medical setting or by personnel that has no access to other alternatives (in undeveloped countries, for example). The use of a reduction maneuver without analgesia might be an alternative in patients with atraumatic dislocations, recurrent dislocations and recent dislocations (within 6 hours); and there are some reports of successful results [1]. Anyway when the patient is evaluated in a medical setting an analgesia technique should be offered to most patients.

### 2.2 Intravenous Analgesia and Sedation

The combination of intravenously administered opioids and benzodiazepines assures muscle relaxation and pain control allowing the practitioner to attain an easy closed reduction. This is often the most commonly used procedure in a medical setting [2] and has been reported to obtain excellent results [2, 3]. It does have some significant disadvantages as there is a significant number of patients who will have secondary effects such as respiratory depression, nausea and thrombophlebitis [4]. This has impact in practice. A qualified assistant is required to control the airway and longer hospital stays can be expected, as the patient has to be monitored until fully alert. As it is generally considered to be the standard of practice it is the most commonly used system to assess the validity of other analgesia methods. Nitrous oxide and oxygen have an analgesic effect and increase the pain threshold. A 50% of N^2^O combination with oxygen (used to assure proper oxygenation) is used frequently in emergency situations when moderate pain is expected. If used according to a defined protocol, it is a safe and effective method to attain analgesia [5]. Its use during shoulder reduction has not been widely reported. One randomized controlled study showed that it had a similar efficacy compared to intravenous analgesia and the time to discharge is significantly reduced [6]. Other studies found relatively poor (10%) [7, 8], because its widespread use cannot be recommended.

### 2.3 Intraarticular Analgesia

The administration of an intraarticular injection of local anesthetic (generally, 10 to 20 ml of lidocaine 1% without adrenaline intraarticularly in a blind approach through the lateral deltoid, 2 cm below the acromion) is a common and simple method to attain some degree of analgesia prior to reduction maneuvers. There have been many randomized controlled trials exploring its efficacy against other anesthetic techniques and that has prompted different authors to perform well designed systematic reviews and meta-analysis [4, 9-12]. The latest, by Jiang *et al.* summarizes nicely the current evidence on the topic based in the results of nine high quality randomized controlled trials that compared intraarticular lidocaine with intravenous analgesia. The success rate was very similar using both systems (79% vs 89%, risk ratio 0.83 to 1.03) but the group managed with intraarticular lidocaine had less side effects (1.7% vs 19.6%; risk ratio 0.04 to 0.32), in particular respiratory depression (0% vs 22.7%, risk ratio 0.02 to 0.26), and had shorter discharge times. No differences were encountered in concern to patient satisfaction orpost reduction pain. As a disadvantage intraarticular lidocaine required a longer interval time between injection to reduction. Intraarticular lidocaine is thus an inexpensive method that can be used in many different settings and that does not require specific patient monitorization or personnel adepts in airway management.

### 2.4 Nerve Blocks

Regional nerve blocks can successfully control pain in many settings. The use of ultrasound guidance during the procedure allows it use in emergency settings. The use of ISNB [13] or SSNB [14] as analgesic techniques for shoulder dislocation reduction has been reported in the literature but little quality information is really available. Blaivas *et al.* in a randomized controlled trial evaluated the benefits of ISNB over intravenous sedation and found no differences in efficacy or side effects but decreased length of stay and decreased one-on-one health care provider time using ISNB [15]. Recently Tezel *et al.* [16]. compared SSNB and intravenous analgesia and found no significant differences except for a longer length of stay in the intravenous group. Anyway, its generalized use in an emergency room setting should be considered with care and should be done by properly trained physicians [17].

### 2.5 General Anesthesia

The use of GA for closed reduction of a dislocated shoulder has significant advantages: it makes the process painless for the patient and facilitates reduction maneuvers because complete muscular relaxation can be achieved. It does have significant drawbacks, because it requires an anesthesiologist and the reduction procedure can be delayed. It is thus not the best option for most shoulder dislocations but is mandatory when other techniques have not been successful in attaining reduction or when a fracture-dislocation is present as performing a manual reduction without complete relaxation might cause further displacement of the fracture [18].

## 3 ANESTHETIC MANAGEMENT OF THE PATIENT WITH CHRONIC SHOULDER INSTABILITY THAT REQUIRES SURGERY

The patient with chronic shoulder instability is usually a younger patient with no comorbidities that require a surgical procedure that rarely exceeds two or three hours; despite of this there are several specific circumstances that apply to this group of patients that have to be taken into account. In the following section we will focus in the preoperative evaluation, intraoperative management and postoperative pain treatment of these patients.

### 3.1 Preoperative Evaluation Formatted:

Most patients undergoing surgery for shoulder instability are healthy adults. However, a full anesthetic preoperative assessment is needed to determine any coexisting medical problem that may contraindicate a particular anesthetic technique. To assess the patient preoperative condition a systematic review searching for possible co-morbidities should be done, with special attention to respiratory pathology. Previous surgeries, like vocal cord surgery, should be investigated for their association with possible recurrent laryngeal nerve damage. Contralateral phrenic nerve damage or contralateral recurrent laryngeal nerve damage are contraindications for many anesthetic procedures.

Any condition must be considered that could lead to hypotension. Many studies have investigated the neurologic complications related to low brain perfusion pressure in shoulder surgery in beach chair position (BCP) [18-20]. These studies show that the choice of lateral decubitus position (LDP) can be better. It is important to evaluate basic laboratory tests (hemogram, hemostasis and basic biochemical values), and chest radiograph only in patients with previous respiratory pathologies like asthma or pulmonary obstructive chronic disease. In the preoperative evaluation, the patient should be adequately informed about the different available anesthetic techniques and the possibility of complications. Sedation, RA, GA, or a combination of them can be done. Before performing a nerve block the patient should be adequately informed in order to minimize patient anxiety and to reduce sedation requirements during the procedure. The choice of anesthetic technique is influenced by the expected surgical duration, the surgical position, patient condition, and patient choice. RA and sedation in a collaborative patient can be suitable for minor procedures, whilst, in more complex or longer procedures the combination of GA with a neural block would be more appropriate.

### 3.2 Intraoperative Management Nerve Blocks

The anesthetic management of patients with shoulder instability could be difficult, by the anesthetic technique, and by patient the positioning during the surgical procedure in the operating room. These procedures were routinely performed in the past with open surgery; now it is possible to avoid the direct trauma in the muscles around the shoulder joint using arthroscopic techniques [21] that lead a better control of postoperative pain [21, 22].

The BP and the cervical plexus cover all the sensitivity of the shoulder joint. BP also provides sensitivity to the anterior chest, the skin area represented by the first two ribs and the posterior triangle of the neck. Generally, the entire joint is supplied by branches of the upper trunk, such as the axillary, suprascapular and subscapular nerves. The musculocutaneous nerve covers the lower part of the shoulder in 30% of patients. The nerve blocks that can be performed as anesthetic techniques for arthroscopic shoulder surgery are mainly ISNB and SCNB. If the latter is performed, an SSNB should be associated because the suprascapular nerve arises from C5 before forming the upper trunk. In the ISNB technique, SSNB is included. The use of ISNB for the intra and postoperative pain management has a Cochrane Database IA recommendation level [23]. Compared with single block, continuous ISNB improves postoperative analgesia after arthroscopic surgery [21].

Sometimes regional techniques must be combined with GA, something mainly determined by the patient position in the operating room. These regional techniques can be performed using traditional equipment, mainly neurostimulation (NE), or ultrasound (US). The US superiority resides in the capacity of exact anatomical identification of the structures for blockade or catheter placement. Additionally, the US improves the technique to perform the blockade and reduce the pain associated with the procedure [21, 23]. At the same time, it is important to remember that ultrasound-guided techniques are always operator dependent, and will only be effective in trained hands. The study of Frederickson *et al.*, compared the catheter insertion procedure performed with NE or US; they found that, in the US group, the reduction of the intraoperative anesthetic consumption was evident, no maneuvers for reposition of the catheter were needed in post anesthesia care unit, and early postoperative opioids requirements were lower compared with the NE group. The number of attempts and subcutaneous needle manipulations were also lower in the US group, and the number of boluses of local anesthetic administered by perineural catheter was lower too (1 US *vs* 4 NE) [24]. The effectiveness and duration of ISNB done by single puncture, not only depend on the suitable use of US group and good performance of the technique; it also depends on the pharmacological type, concentration and volume of local anesthetic used; being preferred mixtures of local anesthetics including short latency and short duration of action, and other with long latency and long duration of action [25]. It is widely known that in the new era of US guided RA, volumes used for performing nerve blocks are much smaller than the volumes used when NE was used. In the case of ISNB small volume like 0,11ml/mm of nerve diameter could be used. Many studies agree that ultralow volumes are clinically effective [26]. In the study of Frederickson *et al.*, five different concentrations and volumes of ropivacaine were administered by an interescalene catheter. Patients were evaluated during the first 24 hours to determine the time interval from the ISNB until the onset of pain. The results whereas evident when the volume or the concentration of ropivacaine was increased, the results were similar: the blocking lasted up to 10 hours [27]. If the blockade is done by single puncture, its duration can be extended by the use of local anesthetic adjuvants, including steroids (mainly dexamethasone), central action alpha agonists (clonidine and dexmedetomidine), adrenaline and neostigmine; being dexamethasone the most commonly used [28].

### 3.3 Patient Positioning

There are two basic positions in which shoulder surgery is performed for instability problems: lateral decubitus position (LDP) or beach chair position (BCP) [29]. The position could be elected by the surgeon mainly considering the surgery type: open or arthroscopic procedures. Independently of the elected position, both surgeon and anesthesiologist should ensure that the bearings are protected to avoid neurological complications; with special attention to head and neck positioning, considering that severe complications could occur: cervical cutaneous neurapraxias, hypoglossal nerve palsies and spinal cord isquemic complications [30]. The choice of the BCP represents a challenge for the anesthesiologist, being the main objective to maintain adequate cerebral perfusion [18]. BCP has been associated with neurologic complications such as stroke, spinal cord ischemia, and blindness. The fact that hypotensive anesthesia is frequently used during shoulder surgery (mainly in arthroscopic procedures) to avoid bleeding, makes the problem more significant [30, 31]. The exact neurological complication mechanism is unknown and there is a great controversy in the tools effectiveness to avoid them. Many etiology factors have been proposed: The inability to assess the real cerebral arterial pressure using peripheral blood pressure measurements; the association of hypotensive and bradycardia due to the malfunction of the usual sympathetic regulatory reflexes; and the malfunction of cerebral self-regulation mechanisms.

If the patient is positioned in BCH position the blood pressure measure can only be done in the contralateral arm (which is commonly used for the intravenous access) or the leg, both located below the head level, this makes necessary some measure adjustment [32]. For each 1,3 cm of height difference, a reduction of 1 mm temporal artery pressure happens [19, 33]. If blood pressure is measured in the lower limbs, the measurements are unreliable, generally overestimating the real blood pressure. Choi *et al.* studied 30 patients during arthroscopic shoulder reconstruction under general anesthesia, noninvasive blood pressure was measured in the non- operated arm and in one of the lower limbs. The results showed that the measured pressure in the leg is higher than that measured in the arm before induction of anesthesia, during induction, before intubation and during beach chair position [34], they concluded that noninvasive blood pressure must be measured in the non-operated arm. In case of ASA III/ IV patients, with associated heart disease or who have major risk factors for cerebrovascular disease, invasive blood pressure measurement placing pressure transducers at the level of the ear should be used [34]. Janssen *et al.* showed the relationship between hypotension and BCP in a double-blind, randomized, prospective study, made in outpatients, half of them anesthetized with GA and ISNB in combination, and the other half only with GA. It showed that GA and ISNB do not increase the incidence of bradycardia or hypotension events that require therapeutic intervention. However, they concluded that the two techniques in combination should be used only in ASA I and II patients [35]. In other studies, however, a higher rate of intraoperative hypotension was evidenced. Trentman *et al.* reported a hypotension rate of 54%, in patients who were receiving antihypertensive medication, operated for shoulder arthroscopic surgery in BCP. The conclusion was that hypertensive patients have higher incidence of hypotension associated with this surgical position [36]. Considering the intraoperative hypotension etiology in patients operated in BCP, a cardiac preload reduction due to decreases in venous return could be an important cause. Kwak *et al.* Evaluated the effect of intermittent sequential compression device in the lower limbs, in BCP for shoulder arthroscopy. The hypotension rate found was 28% in the device group vs 64% in the withou**t** device group [37]; concluding the importance of preload reduction in BCP. The occurrence of hypotensive bradycardia episodes (HBE) seems to be relatively common, appearing in up to 30% in patients under general anesthesia in BCP [33]. These HBE have been defined as a decrease in heart rate of at least 30 beats/min within a 5- minute interval, any heart rate less than 50 beats/min, and/or a decrease in systolic blood pressure of more than 30 mm Hg within a 5-minute interval or any systolic blood pressure below 90 mm Hg [38, 39]. The most common proposed mechanism is the Bezold-Jarisch reflex [40], an inhibitory reflex mediated through cardiac sensory receptors with a vagal efferent limb. In brief, during surgery in the BCP enhanced venous pooling occurs in the lower extremities with a secondary increase in sympathetic tone and ultimately a low volume and hypercontractile ventricle; that leads to an abrupt autonomic withdrawal of sympathetic response and activation of increased vagal tone. This combination of venous pooling and increased vagal tone result in profound bradycardia and hypotension.

The association of epinephrine to the anesthetic agent in an associated ISNB seemed to increase the occurrence of HBE from 4% to 29% in a randomized controlled trial [39]. Prevention of these HBE is desirable and another randomized controlled trial suggested that using prophylactic metoprolol (up to a maximal dose of 10mg to keep the heart rate below 60bpm) significantly reduced the rate of HBE from 28% to 5% [38]. The anesthesiologist should note that HBE are not a preventable complication and that these should be expected, the key being prompt identification of the problem and treatment with intravenous ephedrine. (5 – 20 mg iv). There are complex mechanisms that keep cerebral perfusion in a constant range despite variations in peripheral blood arterial pressure; thus, within a certain range of systemic blood pressures, the cerebral perfusion will remain stable, this is called the self-regulation range [41]. A recent randomized controlled trial that evaluated cerebral oxygen saturation using a near infrared spectroscopy device in both LDP and BCP during shoulder surgery found that self- regulatory mechanisms are impaired in BCP compared to LDP [20, 42, 43]. LDP presents fewer hemodynamic complications because the venous return is not impaired.

To protect the vulnerable face and body areas; improve ventilation and the protection of neurovascular structures placing an axillary roll are the major objectives. The traction on the upper operating arm can damage the brachial plexus. Extreme head rotation position can also damage some cranial nerves [29].

### 3.4 General *vs* Regional Anesthesia

BCP is associated with hypotension and the risk of developing cerebral ischemia. In this position RA and GA in combination can increase the risk of cerebral hypoperfusion [44]. Generally, hypotension is less marked in the patient anesthetized only with blockade, although, a small percentage of them may have hypotension and bradycardia caused by the Bezold- Jarisch Reflex [32, 40]. Jeon *et al.* studied the effect of anesthetics on cerebral oxygenation and systemic hemodynamics in patients operated by arthroscopic surgery in BCP, employing jugular bulb oxygen saturation (SjVO2) and regional brain tissue oxygen saturation (SctO2). For that purpose 40 patients were studied and randomized in two groups, in one propofol was used as hypnotic (P) and in the other nitrous oxide and sevoflurane were used in combination (S/N). The study showed that patients in S/N group had higher values of SjVO2 that patients randomized in the P group. No changes were evident in the regional saturation near infrared spectroscopy (NIRS), which may be due to the direct halogenated vasodilator effect, which increases the ratio of the relationship between arterial blood flow/ venous blood flow [45]. Therefore, the use of halogenated hypnotics in arthroscopic surgery on beach chair position is widely recommended. Another important conclusion is that the measurement of SctO2 should not replaces the measuring of SjVO2 in selected cases [45, 46]. Postoperative management of shoulder procedures is associated with severe pain that might need extensive opioid use. The opioid requirements can be similar to that following thoracotomy, and opioid-only analgesic techniques for shoulder surgery are associated with opioid-related adverse effects such as pruritus, nausea and constipation [47]. “Multi-modal” analgesic approaches incorporating tramadol, non-steroidal anti-inflammatory drugs and paracetamol. Additional evidence has emerged of the adverse effects of both situations: poorly treated acute postoperative pain and acute postoperative opioid use. These effects are opioid-induced secondary hyperalgesia and central nociception-induced central sensitization. These mechanisms can be involved in the pathogenesis of persistent post-surgical shoulder pain [48]. The innervation of the shoulder is complex. With the US imaging is possible to identify the BP branches. Commonly used techniques for postoperative analgesia after shoulder surgery are the ISNB, superficial cervical nerve block (SCENB), SSNB, SCNB, subacromial (SA) block, and intra-articular (IA) injections [49].

### 3.5 Interescalene Nerve Blockade

It is the most commonly used technique for analgesia after shoulder surgery. This block should be done at the level of C6 and the local anesthetic should be injected around C5-C6 nerve roots. Single injection ISNB does not provide a sufficient duration of analgesia after shoulder surgery and needs to be combined with a continuous blockade. ISNB produces a preemptive effect in reducing sensitization of nerve endings after surgical incision, reducing postoperative pain. Compared with single-injection, continuous ISNB improves postoperative analgesia after shoulder arthroplasty and rotator cuff repair [50, 51]. Postoperative pain can be found in the surrounding tissue induced by reflex muscle spasm or by a direct result of surgical trauma. Continuous ISNB compared with single blockade reduces pain, especially with movement, improves sleep quality, reduces opioids requirements, accelerates hospital discharge, rehabilitation and increases patient satisfaction. Compared to continuous infusion alone, a basal infusion with patient-controlled bolus provides betteranalgesia, decreases total local anesthetic consumption and the incidence of breakthrough pain. In a study, with a bolus of 5 ml and a blockade time of 60 minutes, the background infusion rate required to produce an effective analgesia with ropivacaine 0,2% in 50% patients was between 2,8 ml/h and 4,4 ml/h in 95% of patients [52]. Ropivacaine 0,2% provides better analgesia in comparison with ropivacaine 0,1% during the first 24 hours after shoulder surgery [53]. Intermittent bolus regimens are better that continuous infusions only and can be the best way to provide perineural local anesthetic. End- hole catheters are worsens than multiorifice catheters, but with continuous infusion regimens, multiorifice catheters may function as end-hole catheters. In an orthopedic study of analgesic requirements, they find that the cost and placement difficulty systems of continuous local anesthetic infusions are not justified for “moderately painful” surgeries [54].Less painful shoulder procedures can be managed with a single injection ISNB. Considering the patient safety, the catheter placement should be guided by US [55].

Previous studies found the use of interscalene catheters in various settings. Bryan and cols, in a retrospective analysis found a 9,7% prevalence of adverse events [56]. In another study, Marhofer *et al.* found a low rate of adverse events (6,7%), being Catheter dislocation the most common event(3,6%), half of which occurred in the immediate postoperative period and was related with the length of the catheter [57].A longer duration of catheter placement is associated with more adverse effects (catheter dislocations, infections, obstruction) and more hospital re- admissions consequent to these effects.

Diaphragm weakness invariably results from ISNB. Independently of the dose, patients with reduce pulmonary reserve cannot be able to maintain oxygen saturation breathing room air. It is possible to identify the phrenic nerve in 93,5% of patients by US. If it is sought at the level of the cricoid cartilage, this nerve can be indistinguishable from the C5 ventral ramus. Considering the closer anatomy, the ipsilateral phrenic nerve blockade during the procedure is very frequently. One study compared two groups in which different volumes of local anesthetic were administered (5 ml vs 20 ml of 0,5% ropivacaine in a single ISNB), In the low-volume group diaphragmatic paralysis incidence was lower in comparison with the standard volume group (45% vs 100%). Furthermore, low volumes do not guarantee the same block duration [49].

Before the introduction of US, this block was associated with the most incidence of neurological deficits,(related to intracord injection and primary damage of the root by the needle),in second place, being the first place occupied by neurological damages related surgical insult of the brachial plexus [58]. Laryngeal nerve palsy is less common, occurring in 3-6% ISNB [58].

Frequently after 30 minutes of the first bolus. The blockade of these nerves can result in symptoms like hoarseness and dyspnea. Delayed onset vocal cord paralysis after ISNB, particularly in patients with previous neck surgeries and contralateral vocal cord palsy may result in severe airway obstruction [59].

Many patients experienced rebound pain after block resolution, because the analgesia time without catheter is limited to duration of the blockade. It is possible to prolong the blockade time through non- neurotoxical perineural additives. Buprenorphine And clonidine are safe for that purpose at clinical concentrations [49]. The analgesia time increased to a median of 1,7 when ropivacaine 0,75% and dexamethasone were administered together. The mechanism based on the prolongation the of sensory block is not completely understood. It can be partially explained by the direct antinflamatory effect on nerve. Although other studies found that dexamethasone induced perineural vasoconstriction with concomitant slower absorption of local anesthetics [60]. Dexamethasone has multiple effects, such as reducing postoperative pain and postoperative nausea. Intravenous and perineural administration seems to be equivalent in concern to the adjuvant analgesic effect on ISNB [61]. If the dose is increased the analgesic effect is not prolonged [62]. Low doses of perineural dexamethasone, like 4 mg, increases the duration of ropivacaine 0,75%.[60].

Dexmedetomidine has a potent α2adrenoceptor agonist effect, being 8 times more selective than clonidine.

Dexmedetomidine 150 µg if is added to ropivacaine could increase the duration of the nerve block and improve postoperative pain [63, 64].

### 3.6 Supraclavicular Nerve Blockade

This approach must be performed by expert hands because the risk of pneumothorax is too high; this is due to the closer anatomy of the upper chest. It has an incidence between 0,6% to 6,1%.If US is used, and if the first rib and the pleura are identified, this complication is dramatically reduced. A report of 1001 consecutive US guided blockades not complicated with pneumothorax is found [65, 66]. In another large survey of 510 US guided any case of pneumothorax have not been reported [67].

Many studies that have compared computed tomographic scanning and US demonstrate that local anesthetic used for the performance of SCNB spread in cephalic direction between the scalene muscles and the prevertebral fascia. A linear US array transducer is needed to identify the brachial plexus as it passes just posterolateral to the subclavian artery. The blockade must be done just above the clavicle, making the approach in plane, watching the needle all the way, these tips facilitates the process and reduces complications. In a study that included 1169 patients who underwent shoulder surgery and had either an ISNB or SCNB at the discretion of the clinical team, the success rate was excellent for both blocks: ISNB (99,8%) and SCNB(99,4%) [68]. The incidence of complications was low in two groups: hoarseness was less for the SCNB group (22% *vs* 31%) and dyspnea was similar (7% *vs* 10%). In conclusion both blockades (ISNB and SCNB) are equally effective and safe for shoulder arthroscopy. The use of a NE does not improve the efficacy of US in the performance of SCNB.

### 3.7 Suprascapular Nerve Blockade

Almost all the sensitivity innervation of the shoulder joint is covered by the suprascapular and the axillary nerves. If the SSNB is done in combination with axillary blockade can provide an efficacious alternative technique to ISNB for shoulder analgesia when is combined with GA. With this blockade, it is possible to find fewer complications and side effects compared with the traditional ISNB. Although, SSNB also carries a risk of pneumothorax (1-6%) [69]. These blocks can be used for patients with severe chronic obstructive pulmonary disease because the phrenic Nerve is not blocked [49]. The procedure of SSNB consists in a local anesthetic injection in the supraspinatus fossa, with the patient seated and upper limbs pending beside the body. The point of needle introduction should be medial to vertex obtained from two imaginary lines traced over the scapula line and the upper edge of clavicle, laterally to coracoid process. The needle is introduced perpendicularly to skin and advanced in craneo-caudal direction, crossing the trapezium and supraspinatus muscles. The Nerve is located adjacent to the coracoid process [70]. Axillary nerve block is performed following a line that connects the scapula angle to the anterior edge of the acromion and a second line that follows horizontally the scapula Spine, representing at this level the quadrangular space where the Nerve is located. The puncture site can be located in the union of a line that begins in the posterior edge of the acromion and the second line described previously.

In a study of 120 patients that compared an “injection groups” in which ISNB, SSNB, IA are performed and a “control group” managed without blockades. They found lower pain scores in patients randomized in the SSNB and ISNB groups. Patients in the ISNB group presented better pain relief with movement, decreased opioid consumption, and better patient satisfaction compared with patients in the SSNB group. Although, SSNB seems to be superior to IA injection after shoulder arthroscopy [71, 72] .

### 3.8 Subacromial - Intrarticular Anesthesia

These techniques are only effective for arthroscopic procedures, excluding rotator cuff procedures. For open and rotator cuff procedures seem to be slightly better than placebo. The evidence shows the possibility of iatrogenic chondrolysis associated with IA local anesthetic [73], higher with bupivacaine at high doses. Severe complications like catastrophic glenohumeral chondrolysis have been reported. Recently, a study that combined SSNB, axillary block and SA- IA injection found that multimodal shoulder regional analgesic techniques were safe and effective for the management of pain after arthroscopic rotator cuff repair [74]. Ropivacaine seems to be the better choice because it has a safety margin in comparison with bupivacaine The anesthetic injection was done by a single shot around the sub acromial and periarticular tissues.

### 3.9 Superficial Cervical Plexus Blockade

The cervical plexus is formed by the anterior divisions of the upper brachial plexus. The supraclavicular nerves arise from the first fourth cervical nerves and emerge above the sternocleidomastoid muscle. These nerves supply the skin over the deltoid muscle and the posterior parts of the shoulder. A single shot blockade of the brachial plexus, covers the first thoracic nerve roots and the lower brachial plexus. The cervical plexus is not covered thus complete shoulder analgesia cannot be achieved. The same thing happens with ISNB [48]. Superficial cervical plexus blockade US guided can be performed at the midpoint of the posterior frame of sternocleidomastoid muscle, but its use does not increase the success rate of the block compared with a landmark-based technique [75].

## CONCLUSION

A patient with an acutely dislocated shoulder, sedation, general anesthesia or the administration of intraarticular anesthetics constitutes the treatment of choice, because the aim is to recover the normal joint anatomy. Nerve blockades have a limited role in these patients. For surgical procedures in the patient with shoulder instability, the aim is to prevent the dislocation. In these patients usually minimally invasive procedures are performed; thus RA might be the best technique, probably in combination with GA, basically determined by surgical position. Finally, the anesthetic management during shoulder arthroscopic surgery is a challenge for the anesthesiologist. In many cases, the techniques recommended with IA level of evidence cannot be adequately used in our patients, either by previous pathologies of the patients or by inexperience of the anesthesiologist. Also, different complications can occur in relation to the surgical positions used.

## LIST OF ABBREVIATIONS

BCP: = Beach chair position
BP: = Brachial plexus
GA: = General anesthesia
HBE: = Hypotensive bradycardia episodes
IA: = Intra-articular injections
ISNB: = Interescalene nerve block
LDP: = Lateral decubitus position
NE: = Neurostimulation
NIRS: = Regional saturation near infrared spectroscopy
P: = Propofol group
RA: = Regional anesthesia
SA: = Subacromial block
SCENB: = Superficial cervical nerve block
SCNB: = Supraclavicular nerve block
SctO2: = Regional brain tissue oxygen saturation
S/N: = Nitrous oxide and sevoflurane in combination
SSNB: = Suprascapular nerve block
SjVO2: = Jugular bulb oxygen saturation
US: = Ultrasound
